# Landscape-scale conservation design across biotic realms: sequential integration of aquatic and terrestrial landscapes

**DOI:** 10.1038/s41598-017-15304-w

**Published:** 2017-11-06

**Authors:** Paul B. Leonard, Robert F. Baldwin, R. Daniel Hanks

**Affiliations:** Department of Forestry and Environmental Conservation, Clemson University, Clemson, SC 29634 USA

## Abstract

Systematic conservation planning has been used extensively throughout the world to identify important areas for maintaining biodiversity and functional ecosystems, and is well suited to address large-scale biodiversity conservation challenges of the twenty-first century. Systematic planning is necessary to bridge implementation, scale, and data gaps in a collaborative effort that recognizes competing land uses. Here, we developed a conservation planning process to identify and unify conservation priorities around the central and southern Appalachian Mountains as part of the Appalachian Landscape Conservation Cooperative (App LCC). Through a participatory framework and sequential, cross-realm integration in spatial optimization modeling we highlight lands and waters that together achieve joint conservation goals from LCC partners for the least cost. This process was driven by a synthesis of 26 multi-scaled conservation targets and optimized for simultaneous representation inside the program Marxan to account for roughly 25% of the LCC geography. We identify five conservation design elements covering critical ecological processes and patterns including interconnected regions as well as the broad landscapes between them. Elements were then subjected to a cumulative threats index for possible prioritization. The evaluation of these elements supports multi-scaled decision making within the LCC planning community through a participatory, dynamic, and iterative process.

## Introduction

Systematic conservation planning is a fertile and relatively young scientific discipline capable of examining global natural resource threats including land cover conversion, habitat fragmentation, biodiversity loss and climate change. It is primarily concerned with spatially identifying and prioritizing lands and waters important for functioning ecosystems and biodiversity within a transparent planning framework^1,2^. Often, maps of conservation potential represent a balance of social, economic, and regulatory constraints with biodiversity objectives, including both landscape patterns and ecological processes. The planning process, as well as final products, helps practitioners prioritize where and when to take conservation action. However, biodiversity objectives and management actions are rarely captured across realms (e.g., freshwater, terrestrial, marine) as topical but separate analyses are more expedient^3–6^ despite the increasing awareness that cross-realm considerations are essential to managing landscape-level threats to natural resources^7^.

Since landscape-level biodiversity is enhanced by the juxtaposition of different ecosystems, beta diversity is “missed” in conservation plans that consider terrestrial or aquatic conditions in isolation. Cross-realm integration must account for obvious contrasts between systems and across ecotones while maintaining the inevitable linkages that drive diversity^8^. Further, our understanding of local food web interactions is enhanced by considering landscape-level processes that link terrestrial and aquatic realms^9^. This is particularly true in regions such as the Central and Southern Appalachians of the United States where regional aquatic and terrestrial diversity are both high, making the entire region a conservation priority^10^. Within such regions, it remains a challenge to couple divergent ecological realms encountered across broad extents and account for the functional connectivity between and among them^11–13^. Despite recent advances in theory, few synthetic plans and fewer decision-support tools^14^ have overcome these obstacles largely due the complexity of cross-realm interactions and governance^15^. The difficulties are especially apparent when considering large-scale changes (e.g., climate) that threaten functioning ecosystems across realms disproportionately^16^.

To address the large-scale changes occurring on the landscape in the last several decades many conservation planners have employed coarse-filter planning approaches. These approaches focus on ecosystem and evolutionary functions to maintain community-level diversity, in contrast to those approaches that emphasize individual species conservation^17,18^. However strong the support is for geodiversity and other coarse filters underpinning biological diversity, managers are challenged to meet tangible, policy-oriented goals. Methods are needed to achieve target goals across large landscapes that have local applicability, yet still capture large-extent processes^19,20^. Many studies have relied on estimating ecological integrity using a diverse group of conservation target surrogates that can be measured and monitored to indicate ecosystem health^21,22^. Combining coarse-filter data products with finer-grained species distributions that represent important ecological communities is one method of multi-scaled planning. Yet modeled species distributions are fraught with uncertainty when input data are sparse^23^. They add fine scale information to plans when considered at large extents, but fall short of providing decision-relevant information in localities.

Building upon these ideas and recognizing the ‘unprecedented scope of affected landscapes’ from large scale stressors, the United States Department of the Interior created the Landscape Conservation Cooperatives (LCCs) in 2010 to operationalize conservation planning broadly across North America with a focus on mitigating climate change impacts^24^. These 22 self-directed entities are a collection of conservation practitioners from NGOs, tribal, and local, state and federal organizations that share common goals. Using a distributed model that allows divergence based on regional problems, the LCC system has been charged with developing conservation strategies and objectives to maintain natural and cultural resources. Following a review by the National Academy of Sciences^25^, the LCC network built a strategy upon foundational systematic conservation planning principles to guide the development of landscape conservation design projects^26^. Thus, the overreaching goal of this project was to create a regional conservation plan and design for the Appalachian LCC (App LCC) that encompasses many of these principles using the expertise of LCC cooperators to identify a diverse set of regionally significant targets throughout the central and southern Appalachian Mountains. A secondary objective of the project was to explore novel techniques to sequentially integrate aquatic and terrestrial realms in spatial prioritization while loosely-coupling landscape connectivity to better capture biodiversity and ecosystem processes. The final objective was to use science-driven technical outputs and translate them into a conservation design for partner application.

## Methods

### Study Site

The ecologically and socially complex geography of the Appalachian LCC encompasses 592,129 km^2^ intersecting 15 states stretching west from the central and southern Appalachian Mountains towards the interior low plateaus of Indiana and Missouri. Moving westward the major forest cover transitions from oak-pine dominance in the south, maple-beech-birch in the north into a matrix of oak-hickory in the plateaus^27,28^. The ancient geological history, lack of glaciation through many areas, and recent land use history (−12000 years) set the stage for diversity in this region^29^. However, changes in forest cover and topography, due to mining and intense agricultural use over the past few centuries coupled with current forest management (e.g., fire exclusion), have produced a multitude of challenges for landscape conservation^30^. Furthermore, the overwhelming majority of the geography is in private ownership, making it critically important that conservation planning outputs help land trusts, other NGOs, and industry in their private lands conservation efforts.

### Conservation Target Selection, Processing, Goal-setting

Interactive conservation is built upon stakeholder feedback and we employed a participatory approach to select conservation targets and goals with involvement from over 60 regional experts selected from LCC partner organizations. The expert pool was drawn from academic scientists, state wildlife agency managers, and non-governmental organization scientists and conservationists and their participation was focused around two primary themes: (1) subject-area expertise in major taxonomic groups represented by the species of greatest conservation need throughout the region (e.g., freshwater mussels, herpetofauna, birds), and (2) systems-level expertise focusing on physiographic regions (e.g., Piedmont, Blue Ridge, Appalachian Plateaus). A series of ten virtual webinar meetings were held over a period of eight months to interact with expert teams that were divided into three regional subgroups (Central, Southern, and Western) to maximize the opportunity for discussion. Through meeting feedback and follow-up conversations, we selected 26 targets spanning three thematic spatial scales (coarse, meso, and fine) to capture landscape pattern and process. To provide targets added confidence against future change, we projected climate resilience along with energy development and urbanization threats into the year 2030. While it is impossible to successfully model entire ecosystems with measurable benchmarks, many ecosystems can be monitored and modeled using representative targets that are unique or important to those communities (e.g., cove forests, shale barrens)^31^. After a phase of data collection, curation, and revision, we finalized the minimum required goals for each target (i.e., proportion of target represented in conservation plan) with technical team assistance.

Final conservation targets and ancillary data used in the cross-realm planning included landscape-scale processes (e.g., connectivity), species’ distributions, ecological features, and proxies of ecosystem services. Some of these models originated from previously published and/or funded research by the LCC and other analyses were conducted by the authors (S1). For example, the authors created species richness estimates from raw survey data. Data manipulation was performed using a combination of Quantum GIS v. 2.18 (QGIS Dev. Team, OSGF) and ArcGIS v. 10.3 (ESRI, Redlands, CA). For consistency, final targets (see integrated scenario) were summarized at the 1 km hexagon spatial resolution and submitted to Marxan^32^ to find a near-optimal spatial solution that met the targets across the entirety of the study area.

### Spatial Optimization

**Figure 1:**
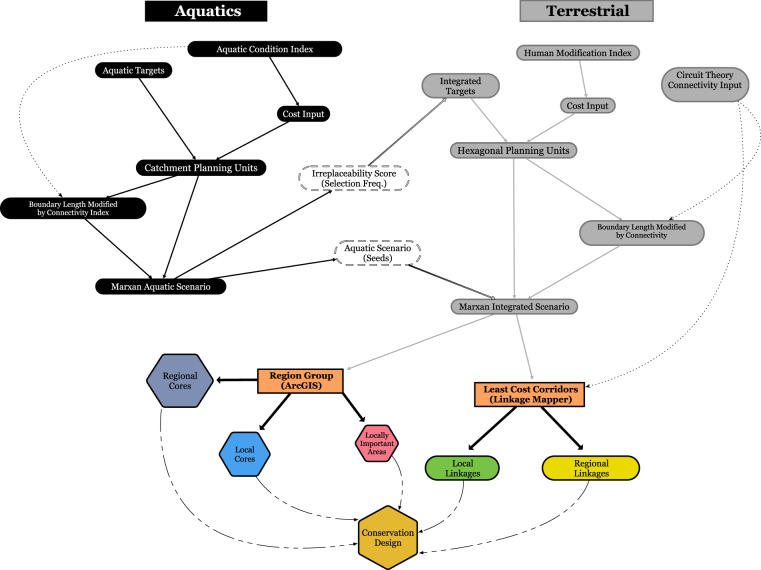
Beginning with the creation of an aquatic condition index, this schematic depicts the workflow of cross-realm integration for freshwater aquatics (black boxes) and terrestrial (grey boxes) ecosystems. White boxes depict how priorities of the aquatics scenario informed the integrated assessment. The integrated scenario feeds into a conservation design phase (colored boxes) where design elements are created. In addition, the marxan aquatic scenario can be used independently to help inform conservation decision-making or feed its own conservation design phase.

In order to integrate planning across realms, we conducted spatial optimization modelling for aquatic conservation targets prior to the terrestrial procedure (Fig. 1). Amis *et al*.^11^ found that influencing terrestrial prioritization with a stand-alone aquatics scenario reliably achieved conservation goals for both, thus increasing beta diversity, while minimizing area. Additionally, this sequence facilitated the use of nested planning units (i.e., catchments inside of larger drainages) that better represent aquatic ecosystems. Planning units for aquatic targets were based on local catchments (i.e., drainage directly into a stream reach) drawn from the National Hydrography Dataset^33^ and were populated with predictors (e.g., water quality, connectivity, flow regime) from a number of sources in order to create a cumulative layer of existing aquatic condition to be used in the optimization analysis as a cost layer (see SI2). For the majority of aquatic targets, we employed boosted regression trees (BRTs) to predict fish and aquatic macroinvertebrate response variables at the network catchment (i.e., drainage area upstream of and into a reach, hereafter, ‘watershed’) scale (Table 1). Boosted regression trees are robust to missing data, have both explanatory and predictive power, and are able to handle complex relationships that are both non-linear and interactive without the need for prior data transformation. By iteratively fitting and combining simple regression models BRT models improve model structure and predictive performance. Tree complexity and learning rate were systematically altered in order to identify the optimal model structure. We reduced model complexity by removing redundant variables (r > 0.80) and variables with minimum variation among sites. Global models were developed with the remaining variables and then further simplified using scree plots of the predictor relative influence. Simplified models were retained if their cross-validation was greater than or equal to that of the global model. BRT models were developed with methods and code provided by Elith *et al*.^34^. In addition, two aquatic targets were created using maximum entropy-based species distribution models^35^ (details on how models were used to create targets are available in SI3). Finally, all targets were used as input to solve Marxan’s minimum set problem, which achieved all conservation target goals simultaneously for the lowest cost (i.e., highest aquatic condition).

**Table 1:** Boosted regression tree results for fish and aquatic macroinvertebrate metrics.

	Fish									Aquatic Macroinvertebrates			
	Richness	Diversity	% Invertevore	% Piscivore	% Herbivore	% Lithophilic	% Coarse Sediment	% Tolerant	% Intolerant	% EPT	% 5 Dominant	% Tolerant	% Intolerant
			Taxa	Taxa	Taxa	Taxa	Taxa	Taxa	Taxa		Taxa	Taxa	Taxa
# of Trees	5250	5950	5600	4700	3850	4600	8150	4550	5650	2150	8300	2700	2600
Learning Rate	0.01	0.01	0.001	0.01	0.01	0.01	0.001	0.01	0.01	0.001	0.001	0.001	0.0005
Total Deviance	6.15	902.95	0.133	18.78	12.57	19.22	6.48	12.85	12.63	2.83	2.27	6.45	1.05
Residual Deviance	2.64	298.56	0.12	8.19	7.8	9.52	4.97	6.12	6.49	1.61	1.68	1.81	0.56
Variance Explained	79%	85%	48%	79%	70%	78%	56%	79%	80%	81%	76%	89%	77%
CV Deviance (se)	8.92(0.08)	433.96(6.15)	0.12(0.03)	11.31(0.22)	10.23(0.22)	12.79(0.17)	5.51(0.23)	8.85(0.31)	10.08(0.26)	2.65(0.32)	2.20(0.25)	2.88(0.35)	0.85(0.12)
CV Deviance Explained	60%	69%	32%	64%	48%	61%	45%	51%	41%	27%	24%	76%	36%
Average thematic % relative influence													
Flow	21.6	15.0	26.3	17.2	21.8	25.2	24.8	17.9	16.4	27.4	14.6	11.0	7.0
Geomorphic	45.9	62.5	48.4	51.5	47.6	38.7	46.3	38.7	39.4	23.4	36.7	6.6	17.3
Connectivity	6.2	4.4	2.3	6.0	6.7	7.0	3.7	10.9	9.3	15.7	13.2	2.3	8.0
Water Quality	8.0	12.0	18.5	9.9	6.7	6.1	7.4	14.6	11.5	12.8	9.2	87.9	56.7
Non-point Pollution	18.2	7.5	7.3	15.7	16.8	22.2	17.5	18.7	22.4	20.9	27.8	6.8	19.2
Point Pollution	1.3	0.6	0.3	1.4	1.4	1.8	1.5	1.8	3.0	1.8	0.0	0.1	1.2

To facilitate the interpretation of individual BRT model results (top) in the context of our thematic framework (bottom), the % relative influence is presented (by theme).  The ‘average thematic % relative influence’ is the percent contribution of each theme to the BRT model. Themes are comprised of flow condition (dam storage, dam density, altered streamflow, agricultural and industrial water withdrawals), geomorphic condition (erosive and resistive forces), connectivity (dam and road crossing density at the watershed and catchment levels), water quality (total nitrogen load, total phosphorous load, and dissolved organic carbon), non-point source pollution (% agricultural land cover, % impervious surface, and % natural land cover in the watershed, active river area, and catchment), and point source pollution (toxic site density, permit compliance system site density, toxic release site density, coal mine, wind turbine and natural gas well density, and mine density).

Planning units for the integrated scenario (aquatics-terrestrial) were based on 1 km hexagons, which were on average smaller than catchments. This scenario minimized the cost for the degree of human modification inside the hexagons^36^ where heavily modified places were more expensive and less impacted places were cheaper. Integrated target models served as input to Marxan much in the same way as aquatic targets but the problem setup differed by incorporating the aquatic optimization scenario in two ways: (1) aquatic selection frequency (importance of individual catchments to solution) was included directly as a conservation target which had to be considered simultaneously with other integrated targets, and (2) the most optimal solution Marxan found in the aquatic scenario was ‘seeded’ into the integrated scenario so that it was the initial landscape considered by Marxan (Fig. 1). To the best of our knowledge, this integration workflow is novel but Amis *et al*.^11^ used a similar methodology to examine co-benefits of cross-realm integration.

### Aquatic Connectivity

Aquatic connectivity was modeled across the entire LCC geography at the catchment and watershed scales. To evaluate aquatic connectivity, we used density of dams and road crossings as these are known to disrupt connectivity within aquatic systems^37,38^. Connectivity data for dams and road crossings were created from the StreamCat database^39^. We multiplied the density of dams and roads, at the catchment and watershed levels, by the relative influence of each connectivity variable in the BRT models and then averaged the connectivity variables. This allowed us to assign a single aquatic connectivity score to each catchment. This aquatic connectivity score was used to modify the boundary relations between catchments so that highly connected catchments were more likely to be selected together in optimization.

### Terrestrial Landscape Connectivity

To model landscape permeability across the entire LCC geography at a spatial grain (270 m) that could inform stand-alone conservation decisions as well as be used in Marxan, we used the circuit theory-based connectivity software gflow^40^. To simulate resistance/permeability to movement for multiple species, we used methods from Leonard *et al*.^41^ to create inputs for connectivity modeling. Inputs were constructed using land cover data, traffic density (AADT), and bridges and underpasses (SI4). We used gflow to calculate a landscape-level solution free of edge effects by buffering the LCC boundary (100 km) and creating random points inside the buffer^42^. These points were connected in a pairwise fashion and the output was clipped to the LCC geography. The landscape connectivity scores were used to modify the boundary relations of hexagons so that highly connected hexagons were more likely to be selected together in optimization. In this way, landscape connectivity can be thought of as loosely-coupled with the optimization algorithm.

### Design Elements and Connectivity

In order to move from optimization outputs to a network design that could be easily communicated and joined with adjacent LCC efforts e.g.^43^, and used to facilitate end-user engagement, we produced five elements with specific conservation functions. Element functions are related to multi-scale processes relevant to decision making both locally and at the landscape scale. (1) ‘Regional Cores’ are broad contiguous areas of landscape-scale significance that are in the near-optimal solution and have high internal connectivity. They were selected using ArcGIS ‘region group’ calculation with a minimum size threshold of 1,000 km^2^. (2) ‘Regional Linkages’ are landscape-scale connectors between regional cores. They were selected using the top 5% of corridors defined by Linkage Mapper v. 1.1^44^. (3) ‘Local Cores’ are smaller contiguous areas of regional significance with high internal connectivity and were selected with a minimum size threshold of 50 km^2^. (4) ‘Local Linkages’ are connectors between local and regional cores and may be nested inside regional linkages indicating redundancy. They were selected using the top 1% of corridors. (5) Locally Important Areas (LIAs) are smaller and often more isolated areas of local significance (although some participate in important corridors) identified by grouping near-optimal hexagons with a minimum size threshold of 5 km^2^. The linkage elements were derived using a similar but more deterministic connectivity metric than gflow called ‘least cost corridors’^45^. However, this method used the same permeability/resistance input as the other connectivity analyses herein and identified pathways that accumulated the least cost to move across the landscape. To compliment the more deterministically derived linkages between the design elements, the finer-grained probabilistic output from gflow can be examined to connect locally import areas and more isolated hexagons selected by Marxan.

### Threats and Prioritization

To place the conservation planning and design exercise into a prioritization framework, we compiled a landscape-level threats analysis. The App LCC geography is known to support some of the richest natural gas and coalfields in the United States and thus energy development is among the top land use change threats in the region. In addition, many sub-regional areas are undergoing rapid urbanization that is likely to fragment habitats for many plants and animals^46^. Moreover, these land use change and intensification trends are likely interacting with changes in climate to produce deleterious effects for biodiversity conservation^47^. We used energy development models produced by The Nature Conservancy that project gas, wind, and coal development into 2030 ^48^. Housing density was projected to 2030 at the census block group scale by researchers at the University of Wisconsin^49^ and NatureServe created a climate exposure index that describes mid-century departure from 20^th^ century baseline climate variability (SI5). Finally, the three landscape-level threats were combined into an additive assessment ranging in intensity from 0–3. A conservative approach was taken to all models where the highest probability of development, climate departure, or housing density (*p* > 75%) was assigned a score of 1 and lower probabilities a score of 0. A simple and intuitive matrix was constructed to compare selection frequency inside design elements from the integrated (aquatics-terrestrial) scenario to compiled threats in order to provide information for prioritization of conservation action.

### Calibration of Optimization

Marxan compares alternative results with an objective function where a lower value is more optimal. There are several variables inside this function that can be adjusted to balance competing goals. We calibrated these variables according to best practice documents^50^. Calibrated objective function variables included: boundary length modifier, individual conservation target penalty factors, and number of iterations. For example, the boundary length modifier (BLM) was calibrated using the Fisher and Church^51^ method to find the ‘sweet spot’ where small changes have disproportionate results on the compactness of solutions. Post-hoc visual inspection and adjustment followed each calibration. Final BLM was set at 0.09375 over 100 repeats of 1.5 billion iterations.

### Assessment of prioritization and conservation targets

We summarized the proportion of individual conservation targets held within the integrated scenario and the design elements versus their global proportions. The proportion of captured targets is a useful way to describe target influence, the degree to which targets capture rare or underrepresented areas (complementarity), and thus a possible framework for prioritizing targets in specific places. The prioritization was broken into 4 categories based on threat and Marxan selection frequency: 1) Low Threat – Low Selection, 2) Low Threat – High Selection, 3) High Threat – Low Selection, 4) High Threat – High Selection. We examined target representation in all five design elements and in the overall design collectively.

## Results

**Figure 2:**
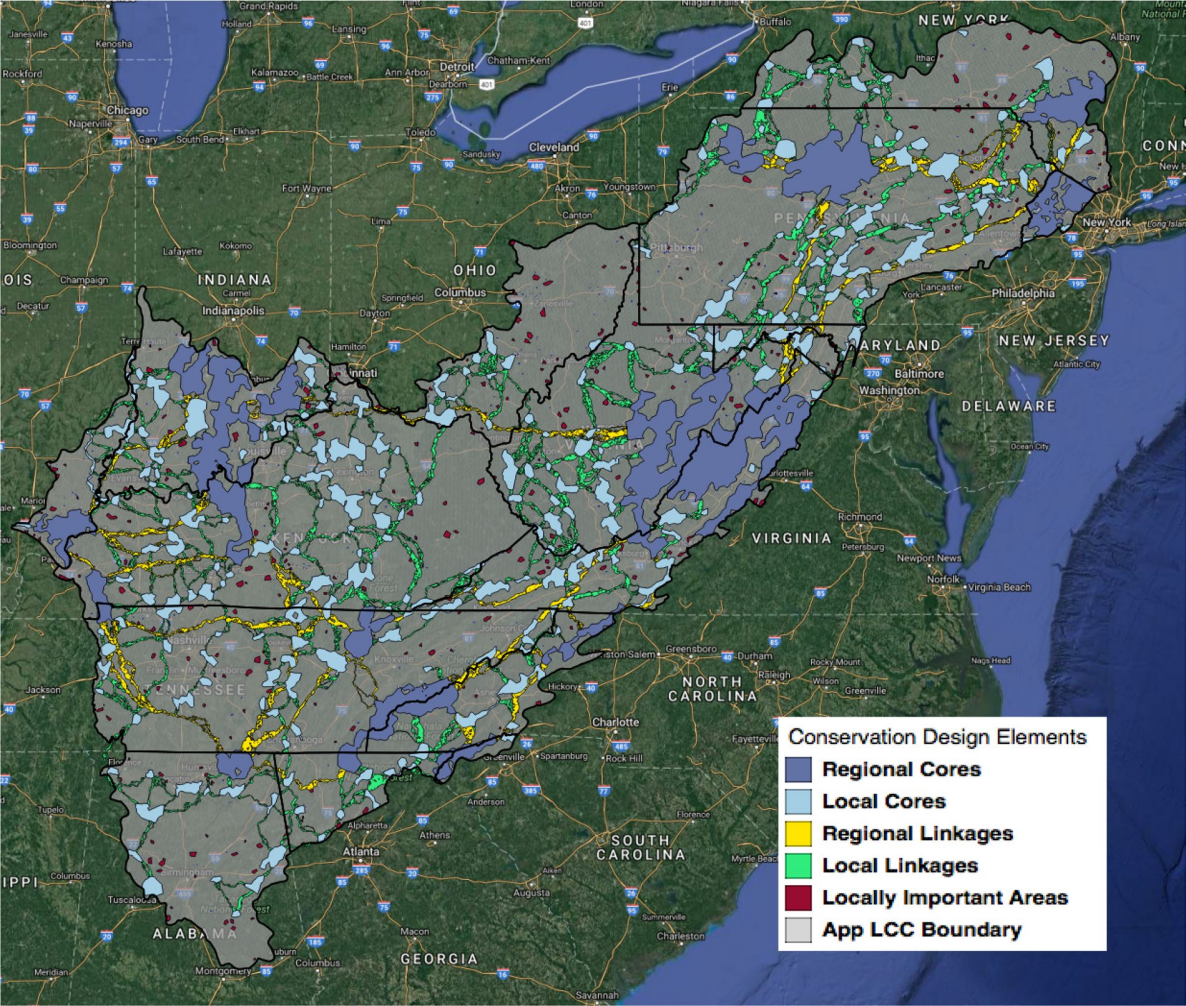
Stylized landscape conservation design for the Appalachian Landscape Conservation Cooperative with all design elements derived from spatial optimization and connectivity modeling. Created with Quantum GIS v. 2.18 ^76^.

Our overall conservation design encompassed a selection of discrete areas that comprised nearly 25% of the landscape (Fig. 2). The 18 ‘integrated’ conservation targets (e.g., both aquatic and terrestrial) were represented disproportionately by this 25% although all target goals were achieved inside optimization. For example, the design captured greater than 50% of the distribution for 14 of the 18 targets. The remaining 4 targets were captured at 49%, 36%, 33%, and 28% of their distributions respectively (Table 2). Two-thirds of the targets captured below 40% were fine-scaled and often cryptic ecosystems (acidic fens and forested wetlands) and the remaining target was a coarse-scale estimate of large, low-lying mature forest blocks. Performance of this target likely suffered since it’s  distribution was limited by elevational gradients and spatially overlaps with other targets, although it performed well in linkage design elements. The five highest performing targets (≥ 65% representation) included climate targets (areas least likely to depart from historical baseline and the nature conservancy’s resilient landscapes), ecosystem service targets (total basal area and carbon storage) and typic montane cove forests. Goals for climate targets were set relatively high and as such, we expected higher levels of representation. However, relatively low goals were set for ecosystem services yet they were important to the optimization (Table 2). We made no distinction between how services were provisioned or regulated and thus their representation was tightly associated with large forest blocks.

**Table 2:** Individual conservation targets within the integrated near-optimal solution with their representation inside individual design elements and within the overall conservation design.

Conservation Target	Regional Cores	Regional Linkage	Local Core	Local Linkage	Locally Important Areas	Total	Percent Coverage in Design of Total Distribution
	Area (km^2^)						
Acidic Fens	89.86	71.99	27.30	35.30	14.72	239.17	28.25%
Total Basal Area (Basal)	2274.27	940.15	492.21	791.13	77.57	4575.33	71.47%
Carbon Storage (Carbon)	1894.91	346.84	207.50	288.07	23.11	2760.42	73.13%
Cave Obligates (Terrestrial)	19232.68	41195.88	195.56	160.71	9199.08	69983.91	76.34%
Climate Departure (Climate)	5181.59	1893.80	1317.74	1652.64	117.88	10163.65	84.22%
Forest Importance for Drinking (F2F)	9038.06	11976.62	6452.61	9678.74	137967.37	175113.40	64.43%
Forested Wetlands (F. Wetlands)	779.51	876.50	467.64	574.76	199.87	2898.29	32.75%
Golden-winged Warbler (GWW)	11874.19	6056.68	3960.17	5578.35	592.04	28061.43	64.53%
Irreplaceable Aquatic Areas (I. Aquatics)	17036.28	16113.02	3186.14	4516.45	2033.32	42885.21	54.03%
Lowland Mature Forest (LMF)	1503.08	1914.17	1202.57	2696.30	255.98	7572.10	35.60%
Red Spruce	4228.11	674.95	414.64	486.07	88.81	5892.57	61.77%
Resilience	6054.56	4868.85	2678.64	3198.41	391.04	17191.50	67.38%
Rich Montane Cove Forests	290.89	65.92	35.46	43.23	14.05	449.54	62.27%
Rocky Outcrops	2971.91	1199.89	849.14	942.26	175.24	6138.43	53.24%
Shale Barrens	257.13	97.39	37.79	72.02	4.01	468.33	48.97%
Spotted Skunk (eastern)	22109.16	13356.96	8413.15	13835.31	1429.79	59144.36	57.53%
Typic Foothill Cove Forests	1231.43	590.55	260.05	525.29	74.69	2682.01	49.36%
Typic Montane Cove Forests	1484.67	369.86	285.55	529.78	52.70	2722.56	64.99%

**Figure 3:**
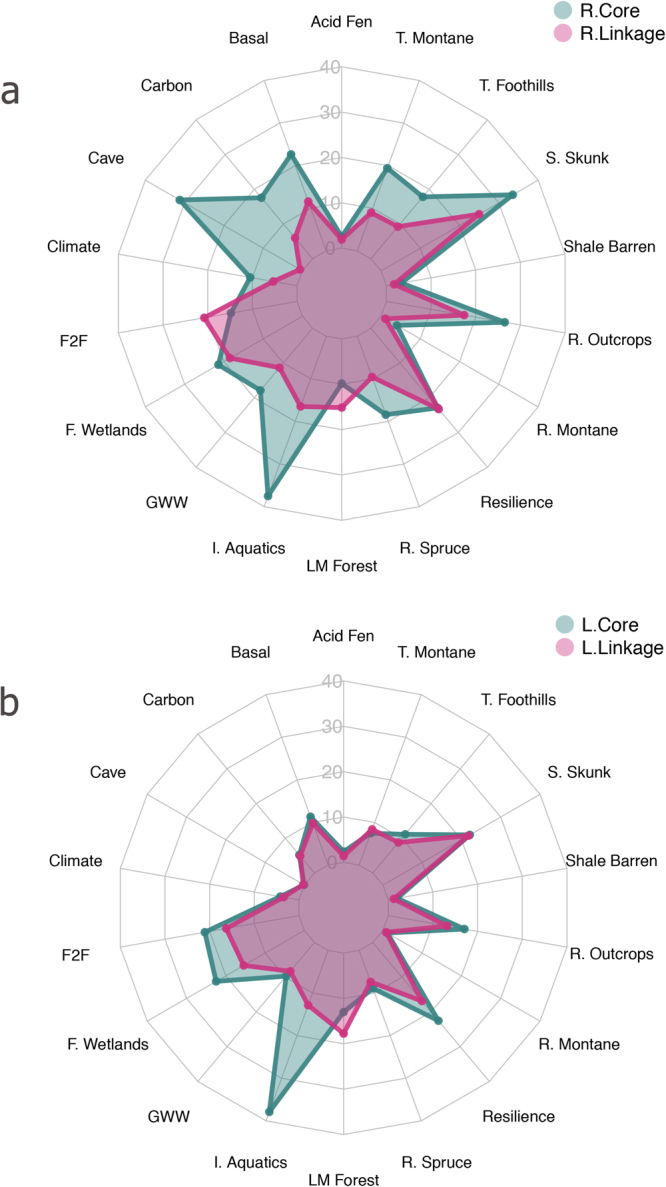
Percent occurrence of 18 integrated conservation targets within planning units captured by two coarse-scale design elements (**a**) Regional Cores and Regional Linkages and those same targets captures by finer-scale design elements (**b**) Local Cores and Local Linkages). Abbreviated targets include Total Basal Area (Basal), Carbon Storage (Carbon), Cave Obligate Density – Terrestrial (Cave), Climate Departure Index (Climate), Forest Importance for Drinking Water (F2F), Forested Wetlands (F. Wetlands), Golden-winged Warbler (GWW), Irreplaceable aquatic areas (I. Aquatics), Lowland Mature Forest (LM Forest), Rich Montane Cove Forests (R. Montane), Red Spruce (R. Spruce), Rocky Outcrops (R. Outcrops), Spotted Skunk (S. Skunk), Typic Montane Cove Forests (T. Montane).

The functional design elements captured multi-scaled effects from conservation targets. At the coarsest spatial scale, regional cores (n = 15, $\bar{x}$ = 3,987 km^2^, σ = 4,979 km^2^) were made up of large contiguous groupings of targets (e.g., cave/karst obligate species, carbon storage, basal area, and irreplaceable areas for aquatics). Regional linkages (n = 18, $\bar{x}$ = 1,313 km^2^, σ = 1,544 km^2^) captured a unique suite of landscape-level targets that were less prevalent inside the cores (Fig. 3a). The forest importance to drinking water, low-elevation mature forests, and resilient landscapes targets had greater representation in coarse-scale linkages than in regional cores (+6%, +5%, and +1%, respectively). At finer-spatial scales the local cores (n = 242, $\bar{x}$ = 209 km^2^, σ = 190 km^2^) captured irreplaceable areas for aquatics, forested wetlands, and patchily distributed early successional targets such as spotted skunk and rocky outcrops (Fig. 3b). Similarly, the spatially dense local linkages (n = 3,126, $\bar{x}$ = 12 km^2^, σ = 46 km^2^) contained higher representations of low-elevation mature forests and mid-elevation typic montane cove forests than local cores respectively (+5% and +0.2%). These linkages connected cores in the southern Blue Ridge and in the Ridge and Valley provinces of the central Appalachians. Our finest-grain elements, locally important areas (n = 379, $\bar{x}$ = 16 km^2^, σ = 12 km^2^), appeared to function in two primary ways: (1) LIAs acted as buffers around existing protected areas suggesting that many conservation values around the protected area were not fully protected; and (2) small areas that had unique conservation value regionally but were not protected.

**Figure 4:**
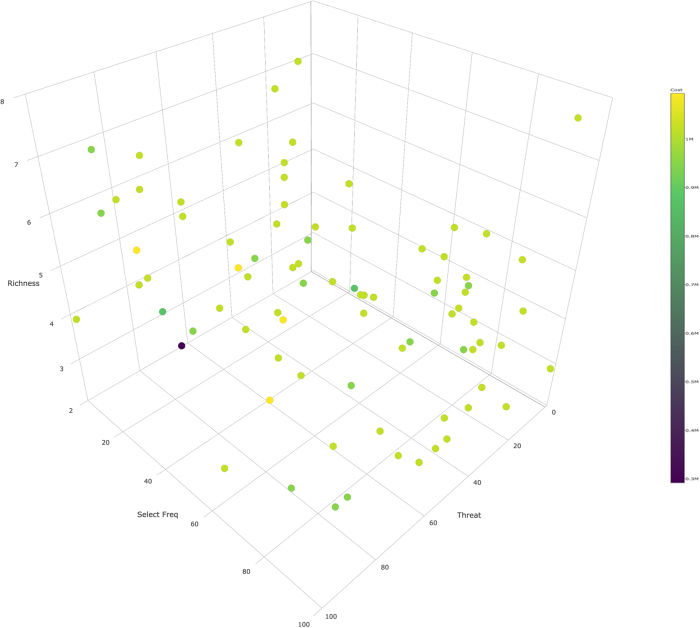
Points represent 100 randomly sampled planning units from the integrated optimization. Planning units with high selection frequency (y) and high threat (x) typically receive a high conservation priority. However, alternative sites may be considered using several additional axes. Two examples are provided herein, planning unit target richness (z) and cost of planning unit (color).

Once conservation opportunities are identified they may be placed into a multi-scaled prioritization framework that facilitates collaborative work within the conservation community. Relative to the 25% of the study area covered by the design elements, only 4.5% of was found in category 4 of the prioritization framework (high threat score and high selection frequency from Marxan). Conservation targets in this category ranged from 1.5% representation (rich montane cove forests) to 39% representation (irreplaceable aquatic areas). Category three (high threat score and low selection frequency) areas constituted only 2.5% of the design area. The most common prioritization categories were assigned to the lower threat areas within the design regardless of selection frequency (93%), although we recognize there are scenarios where practitioners will prefer to prioritize solely on selection frequency. Similar in magnitude, category 4 linkages comprised 1.5% of all linkages in the design compared with 5.5% in category 3. Since all planning units in the design are important, our prioritization framework, similar to Margules and Pressey^2^, focuses on possible alternatives to high value sites regardless of how practitioners choose to rank the categories. (Fig. 4). However, we propose additional ways to interpret alternatives within this framework (e.g., individual design element membership, connectivity score, overall planning unit target richness and cost of planning unit) that provide multiple axes of information.

## Discussion

We developed a landscape-scale conservation plan and design that encompasses some of the most biodiverse aquatic and terrestrial ecosystems in North America^52,53^. Many of these ecosystems are of global significance and are experiencing increased pressure for land-cover conversion. These stressors coupled with climate change are creating complex challenges for conservation planners^53^. While local planners rightfully focus on proximal problems, many of the same problems are occurring at increasing spatial scales and need to be addressed across nested scales and broad geographies. Using appropriate science, landscape cooperatives and alliances (e.g., the LCC system in the US) have an opportunity to offer a unifying conservation planning vision to local, regional, and national planners. However, the success of these efforts may largely depend on stakeholder investment and collaboration^54^. For example, another study within our project area found that social drivers have been more important than ecological ones in predicting private lands conservation to date^55^. Since greater than 90% of the land is in private ownership, we used a broad participatory process to identify conservation targets. These targets were then used as indicators of ecosystem processes, integrity, and resilience and together we set goals around these targets to capture an efficient portfolio of lands and waters. It will be critical for the partnerships built around LCCs to help achieve the goals.

Spatially-explicit areas identified by this study represent a systematic conservation approach to collective LCC conservation priorities. We overcame several off-the-shelf limitations of Marxan software (e.g., cross-realm planning units, connectivity) by complimenting the analysis with both circuit-theory based^40^ and least cost corridor connectivity analyses^45^. We also combined aquatic-based planning units and aquatic connectivity components into the integrated optimization framework^11,15,22^. Our process identified roughly 25% of the overall geography to accomplish the conservation goals (SI1). This coverage is similar to other large-landscape efforts^43,56,57^ although goals for our individual targets varied between 5% and 50%. A review of 159 conservation plans found an overall mean goal of 30%^57^ but individual target goals are often set by expert opinion and typically cover a broad range that accounts for conservation need and distribution size^58^. While it is challenging to answer the ‘how much is enough’ question for every species and system, this research followed the planning best practices of representation, redundancy, and resilience that support the persistence of evolutionary potential^59^.

Patterns of target importance on the landscape indicated that aquatic and ecosystem services targets were important additions to the design. For example, the forest importance to drinking water target was well represented across design elements and spatial scales. Egoh *et al*.^60^ found that only 8% of conservation planning studies included an ecosystem services component between 1998 and 2005 despite the strong and growing awareness of the importance of the services and their relationship with biodiversity^61^. Other targets appeared less efficiently represented in our design either by overrepresentation or the inability to distinguish high priority and/or underrepresented areas. For example, targets representing early successional habitats (e.g., spotted skunk, golden-wing warbler) commonly co-occured with other targets, thus having less influence on the overall optimization. We suspect the species distribution models used to represent these targets suffered from high levels of type I error (commission) due to the more dynamic nature of these systems. Further, these errors are known to vary across elevational gradients^62^ and optimization is less efficient when these errors occur in models of rare species than in more common species^63^. Conversely, several targets consistently influenced optimization by identifying spatially unique areas (e.g., cove forests and both climate related targets). These results align nicely with other studies that suggest incorporating climate diversity, refugia, and corridors into systematic planning, make the conservation plans more robust to uncertainty^59,64,65^.

A novel part of this study includes the integrative nature of terrestrial and aquatic planning units in a sequential optimization that accounts for loosely-coupled, cross-realm connectivity. There has been an increasing call for cross-realm integration as spatial decision-making becomes more common in real-world planning^7,66^. Our methods may be useful to other large-landscape planning efforts that need to incorporate the interacting effects of climate and land use change on viable populations and biodiversity over time. In the future, we suggest a refinement of these methods by nesting conservation planning efforts inside the regional cores. This would allow for localized data at finer-resolutions to be included and assessed through the lens of local governance obstacles that may aid in implementation of the conservation plan^6,12^.

In the integrated solution we used Marxan to optimize 1 km hexagonal planning units and thus, some of the fine-scale heterogeneity in target data was smoothed. This spatial resolution was chosen to balance fine and coarse grain data availability, with considerations for implementing the conservation plan, and limitations of the software itself. Although other large-landscape plans have varied in spatial resolution from 500 m^67^ to 10 km^68^, our 1 km hexagons likely masked important fine-scale information (e.g., zones of interaction between targets) in topographically complex areas. In addition, we forced linkages between core areas identified by optimization and linkages themselves did not account for climatic envelopes. In the future these limitations could be lifted by tightly-coupling spatial optimization with connectivity. Our target data varied widely in spatial resolution and quality as many state and regional datasets were compiled to accommodate full coverage of the study area. Another limitation of Marxan is the inability to explicitly account for data quality. Using best guidance from technical teams, we accounted for uncertainty in data implicitly through goal-setting where data with less confidence often, but not always, received lower goals. We suggest data quality be one of the initial drivers in target selection unless there is to be a new data creation/acquisition phase. In cases where data quality limits the influence of targets on optimization, we suggest an additional target-setting feedback loop after initial examination.

The two primary areas for improvement to our results are in data quality and the development of new analytical techniques. The most immediate contribution could be made to the aquatic condition assessments through improved data on aquatic macroinvertebrates. Data to refine our models were lacking in large regions of the geography and we likely underestimate relationships between aquatic condition and aquatic communities. In addition, rare ecosystem representation could be enhanced through data sharing among LCC partners, specifically for endemic plant communities that were important to many stakeholders throughout the region. The second area involves tightly-coupling connectivity with cross-realm planning units. Although evidence suggests our sequential integration is an improvement on separate realm planning, the optimization algorithm is likely less efficient. The conservation design itself and derivate products are meant to represent the collective priorities of partners around the region. As such, products can provide a regional prospective to local and/or state planning. Practitioners may choose to consider these products as one of many pieces of data to inform a conservation decision. Since conservation planning science benefits from the advancement of data acquisition technology and increasing spatial grain, along with our ability to manage and analyze data^69^, this should be considered a living plan subject to regular updates.

Large-landscape conservation planning science offers a new paradigm under which to analyze conservation decisions across spatial and temporal scales. With modern planners scaling up their thinking about how human impacts will influence future landscapes, bolder and bigger thinking is required to implement these plans^70–72^. Landscape-level cooperatives and alliances can bring together spatial modelers, ecologists, biologists, land use planners, land trusts, and public land managers who can work together in a conservation planning and design exercise to accomplish region-wide biodiversity conservation. Because roles and responsibilities of the players involved in such exercises, along with shifting political and economic realities, are highly dynamic, planning must remain iterative and amenable to new conceptual frameworks and data in order to be successful^73–75^.

### Data accessibility

Model output data as well as conservation design output are available through the Appalachian LCC’s data portal here: https://applcc.databasin.org/ and https://www.sciencebase.gov/catalog/folder/5947e765e4b062508e34424a.

## Electronic supplementary material

Supplementary Information
