# Supplementary material for: Landscape-scale conservation design across biotic realms: sequential integration of aquatic and terrestrial landscapes
Source: Sci Rep. 2017 Nov 6;7:14556. doi: 10.1038/s41598-017-15304-w (PMC5673968; doi:10.1038/s41598-017-15304-w)
Supplement: Supplementary file 1 — Supplementary Information [file 41598_2017_15304_MOESM1_ESM.pdf]

Landscape-scale conservation design across biotic realms: sequential integration of aquatic and terrestrial landscapes

Paul B. Leonard[1], Robert F. Baldwin[1], R. Daniel Hanks[1]

[1]*Department of Forestry and Environmental Conservation, Clemson University, Clemson, SC 29634, USA*

Correspondence:

Paul B. Leonard*: pbleona@clemson.edu

261 Lehotsky Hall. Clemson University 29634: Phone – (803) 524-4209

Robert F. Baldwin: baldwi6@clemson.edu

R. Daniel Hanks: rhanks@clemson.edu

SI 1. Integrated conservation targets used in the landscape conservation design for the Appalachian LCC. Target notes include processing details on how goals were set from individual models (e.g., ecosystems, species, climate). Sources cited as ‘Authors’ include the authors of this manuscript.

Target	Source	Scale	Goal (Prop)	Goal Achieved	Target Notes
**Acidic Fens**	Authors	Fine	5%	100%	Filtered from NWI, Ecological Classifications
**Total Basal Area**	(77)	Coarse	15%	100%	Only scores in top quartile used
**Carbon Storage**	(78)	Coarse	15%	100%	Only scores in top quartile used
**Cave Obligates (Terrestrial)**	(79)	Meso	40%	100%	Group Richness
**Climate Departure (Inverse, e.g., resilience)**	Nature Serve. See SI 2	Meso	35%	100%	Lowest quartile used; places least likely to change from historical baseline
**Forest Importance for Drinking**	(80)	Coarse	15%	100%	Only scores in top quartile used
**Forested Wetlands**	(81)	Fine	20%	100%	NWI: minimum size of 1 acre. PFO class.
**Golden-winged Warbler**	(82)	Fine	5%	100%	Only top half of probability used
**Irreplaceable Aquatic Areas**	Authors	Coarse	50%	100%	Selection Frequency of top 80% used from Aquatics only prioritization
**Lowland Mature Forest**	Authors	Coarse	15%	100%	Filtered from NLCD 2011. > 75% forest cover below 650 m elevation. Min. size 1km
**Red Spruce**	(83)	Fine	20%	100%	Only top half of probability used
**Resilience**	(84)	Meso	35%	100%	Only scores >= 1 sd above the mean used
**Rich Montane Cove Forests**	Authors	Coarse	35%	100%	Filtered from ecosystem classification models and Keener 1983
**Rocky Outcrops**	Authors	Coarse	25%	100%	Filtered from ecosystem classification models
**Shale Barrens**	Authors	Coarse	5%	100%	Filtered from NWI, Ecological Classifications, Elevation thresholds
**Spotted Skunk (eastern)**	Authors	Coarse	5%	100%	Species Distribution Model; top half of probability used
**Typic Foothill Cove Forests**	Authors	Coarse	35%	100%	Filtered from ecosystem classification models and Keener 1983
**Typic Montane Cove Forests**	Authors	Coarse	35%	100%	Filtered from ecosystem classification models and Keener 1983

SI 2. Thematic framework and predictor variables used in assessment of aquatic condition. Datasets without a source were produced by the authors of this manuscript. The Network Catchment (NC) scale for this assessment should be thought of as the watershed scale, which includes the drainage area upstream of catchments.

Themes	Predictor variables	Scale	Comment	Source
General	Catchment area	Catchment		85
	Upstream Drainage Area (Net Catch Area)	NC	Downstream End of Flowline	85
Flow Regime	Base Flow	Catchment		85
	Base Flow	NC		85
	Recharge	Catchment		85
	Recharge	NC		85
	Total Storage/mean annual flow	NC		85
	Density of Large Dams	Catchment		39
	Density of Large Dams	NC		39
	Municipal Water Withdrawal	HUC12		86
	Agricultural Water Withdrawal	HUC12		86
	Industrial Water Withdrawal	HUC12		86
Connectivity	Density of Dams Upstream	Mainstem		86
	Density of Dams Downstream	Mainstem		86
	Density of Road Crossings (Tiger 2010)	Catchment		39
	Density of Road Crossings (Tiger 2010)	NC		39
Water Quality (Human)	Anthropogenic Nitrogen	NC		86
	Anthropogenic Phosphorus	NC		86
	Anthropogenic Sediment Yield	NC		86

Water Quality (Land use)	Impervious (NLCD 2011)	Catchment		85
	Impervious (NLCD 2011)	NC		85
	Impervious (NLCD 2011) Active River Area	Catchment		
	Impervious (NLCD 2011) Active River Area	NC		
	% Natural (NLCD 2011) Active River Area	Catchment		
	% Natural (NLCD 2011) Active River Area	NC		
	% Agriculture (NLCD 2011) Active River Area	Catchment		
	% Agriculture (NLCD 2011) Active River Area	NC		
	% Agriculture (NLCD 2011) Active River Area	Catchment		
	% Natural (NLCD 2011)	NC		85
	% Natural (NLCD 2011)	Catchment		85
	% Agriculture (NLCD 2011)	NC		85
	% Agriculture (NLCD 2011)	Catchment		85
Water Quality (Pollution)	CERC	Catchment	#/catchment area	86
	Permit Compliance	Catchment	#/catchment area	86
	Toxic Release Sites	Catchment	#/catchment area	86
	Coal Mine Density	Catchment	#/catchment area	86
Other	Temperature	Catchment	mean annual temp July	86
	Temperature	Catchment	mean annual temp Aug	85
	Elevation	Catchment		85
	Elevation	NC		85

	Slope	Catchment		85
	Slope	NC		85
	R-factor	Catchment		85
	R-factor	NC		85
	K-factor	Catchment		85
	K-factor	NC		85
	Sand	Catchment		85
	Sand	NC		85
	Silt	Catchment		85
	Silt	NC		85

SI 3. Aquatic conservation targets and goals used in the landscape conservation design for the Appalachian LCC. Target notes include processing details on how goals were set from individual models (e.g., ecosystems, species). Sources cited as 'Authors' include the authors of this manuscript. Each of the predicted responses from boosted regression tree models were separated into a 'high', 'medium', and 'low' category based on 20% quantiles and received their own goals accordingly. Aquatic cave obligate density was separated into 4 categorical, equal interval categories from the entire distribution.

Target	Source	Goal (Prop)	Target Notes
Combined Macro-Invert Score	Authors	multiple	5% low; 10% mid; 35% high
Combined Fish Score	Authors	multiple	5% low; 10% mid; 35% high
Intolerant spp. Score	Authors	multiple	5% low; 10% mid; 35% high
Tolerant spp. Score	Authors	multiple	5% low; 10% mid; 35% high
Fish Diversity Score	Authors	multiple	5% low; 10% mid; 35% high
Aquatic Cave Obligate Density	(79)	multiple	10% low; 30% mid; 40% high; 50% highest
Brook Trout (*Salvenlinus fontinalis*)	(87)	50 %	Species Distribution Model; top half of probability used
Eastern Hellbender (*Cryptobranchus alleganiensis alleganiensis)*	Authors	20 %	Species Distribution Model; top half of probability used

SI 4. The following tables include information to produce resistance surface used in probabilistic (GFlow) and deterministic (Linkage Mapper) landscape connectivity modeling. Landcover classes (A) and traffic density on roads (B), along with reduction in resistance values (C), for more permeable features, were compiled to achieve a seamless resistance layer. Reductions (C) reduce resistance of land cover classes (A). Since there is not a database of wildlife crossings in the southeastern United States, we made conservative estimates for permeability. We digitized several known structures and others from aerial photographs without data on animal movement.

A.

Land Cover Class	Resistance
Open Water	41
Open Space Developed	50
Low Intensity Developed	44
Medium Intensity Developed	64
High Intensity Developed	88
Barren Terrain	41
Deciduous Forest	2
Evergreen Forest	5
Mixed Forest	3
Shrub/Scrub	8
Grass/Herbaceous	25
Pasture/Hay	29
Cultivated Crops	25
Woody Wetlands	13
Emergent Herbaceous Wetlands	20

B.

Traffic Range (Vehicles/day)	Resistance
0-500	2
500-1,400	9
1,400-5,000	25
5,000-14,000	50
14,000-35,000	73
35,000+	89

C.

Category	Resistance Reduction
Railroad Bridges	-40%
Pedestrian Bridges	-30%
Other Bridges	-50%
Water Bridges (8-20m)	-60%
Water Bridges (over 20m)	-40%
Wildlife Crossings	-10%

SI 5. Climate Change Exposure Methods prepared by NatureServe for the Appalachian Landscape Conservation Cooperative.

Climate Exposure

Quantifying projections of climate change exposure across a landscape is a key component in realizing and managing vulnerability. Climate change exposure is defined as the nature and magnitude of climate change that a species, system, or landscape may experience [88]. For the Appalachian LCC, we generated a single multivariate index of mid-century climate change exposure for use in a Marxan conservation prioritization modeling effort being undertaken by Clemson University. The exposure metric chosen for this research describes mid-century future departure from 20th century baseline climate variability. Using historical variability as a baseline to measure the magnitude of projected change is more ecologically relevant (than calculating change in degrees or mm) because inter-annual variability is related to species ecological resilience and therefore changes outside this range should be highlighted [89,90,91].

Mahalanobis Distance

The statistic used to characterize exposure is Mahalanobis distance, which is similar to previous distance metrics used in climate change exposure mapping [92, 93]. Mahalanobis distance is a dissimilarity metric that represents magnitude of change for a set of climate variables, relative to their baseline variability. This metric also incorporates the correlation between those variables by calculating the principal components of the input climate dataset before calculating a standard Euclidean distance. By incorporating correlation between variables, this metric highlights areas in the Appalachian LCC that are outside the range of variability – in terms of magnitude of change as well as novel combinations of climate variables. This metric is calculated for each 1km pixel across the study area and then mapped characterizing spatial variation of climate change across the landscape.

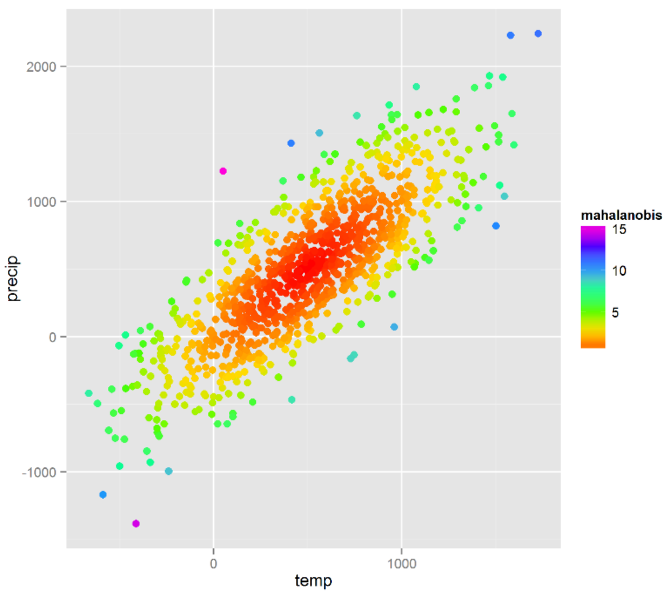

Figure 1. Hypothetical 2-dimensional illustration for Mahalanobis distance calculation for one pixel, showing Mahalanobis distance in color where each of the points represent one year in a time series. The red points in the center are years that were typical of the baseline climate and have a low Mahalanobis distance index. Magenta points have the highest values and represent an unusual departure from the baseline mean.

Climate Data

Climate exposure was measured using the Climate North America dataset downscaled at 1km resolution, which includes historical and projected future time series from 1901-2100 [94]. The climate variables chosen to be included in the multivariate exposure metrics were mean annual temperature (MAT) and annual climate moisture deficit (CMD). Climate moisture deficit is derived from the sum of the monthly difference between atmospheric evaporative demand and precipitation and is used as an indicator of drought [95].

The baseline period used to measure change was a 30-year average of 1950-1979. This time period was chosen because historical weather station density is higher during this time period, reducing error and uncertainty in interpolated climate surfaces. Analysis of future change was based on an ensemble of 15 general circulation models (GCMs) from the latest IPCC 5th Assessment Report [96], for the mid-century projection (2041-2070, referred to as the 2050s) for one emission scenario (representative conservation pathway 4.5). RCP 4.5 is considered an intermediate emissions scenario with a mean global temperature increase of 1.5 degrees Celsius.

Climate Change Exposure Index

Mid-century change is projected to exceed baseline variability for a significant portion of the Appalachia LCC study area. The climate change exposure index shows areas of low elevation to be highly exposed to climate change. Areas of low exposure are highlighted in the Valley and Ridge in West Virginia and southern Blue Ridge mountains in Eastern North Carolina.

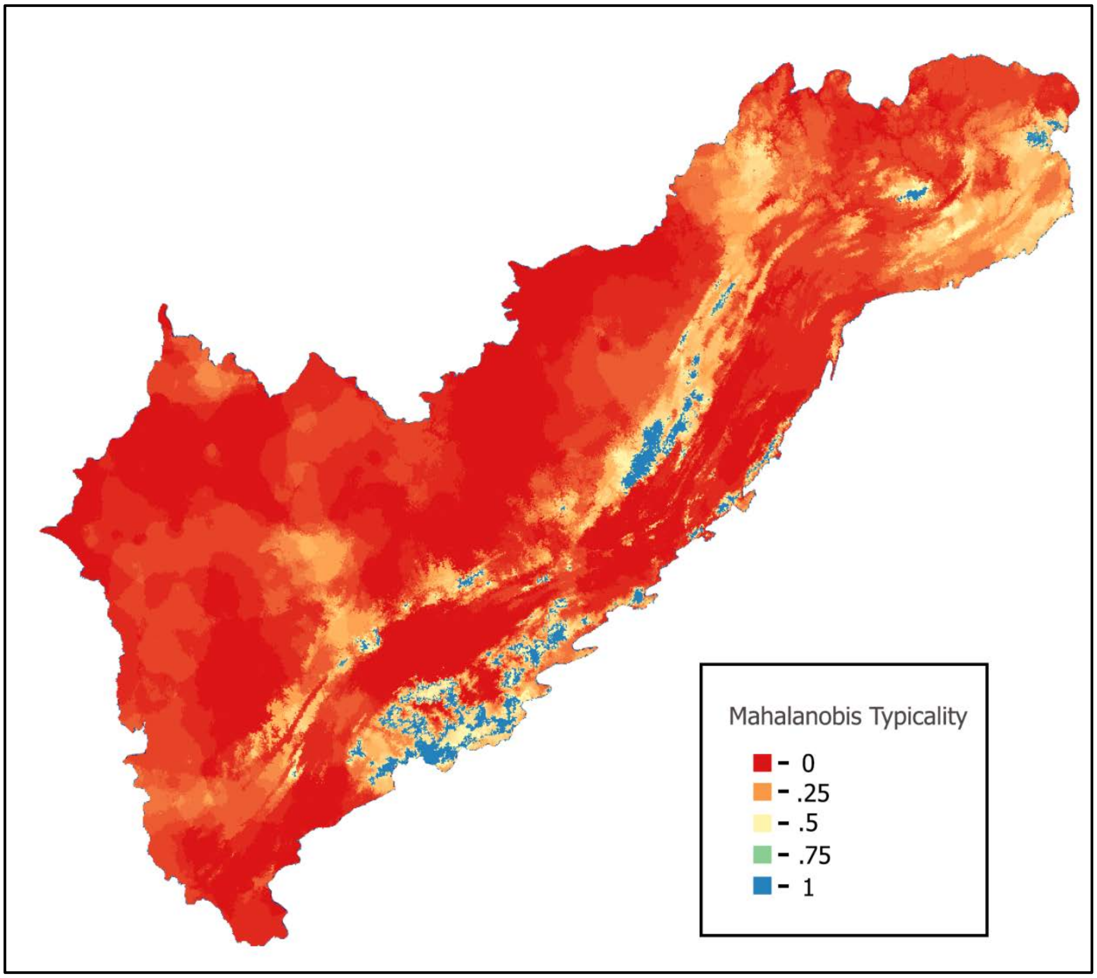

Figure 2. The above panel displays the climate exposure index for the Appalachian LCC using mean annual temperature and climate moisture deficit. To make this index more interpretable we converted it to a 0-1 percentile. We calculated the Mahalanobis distance between the baseline mean value and each individual year in the baseline (1950-1979). Then we calculated the percentile of the Mahalanobis distance for the future mean (2050 under scenario RCP 4.5) relative to baseline. Values between 0-1 describe the proportion of years in the baseline that were more extreme than the future time period where low values represent a high departure from baseline and higher values represent little or no change. Created with Quantum GIS v. 2.18 [76].

References

77. Woudenberg, S. W., B. L. Conkling, B. M. O'Connell, E. B. LaPoint, J. A. Turner, and K. L. Waddell. 2010. The Forest Inventory and Analysis Database: Database description and users manual version 4.0 for Phase 2.
78. Woodall, C. W., J. W. Coulston, G. M. Domke, B. F. Walters, D. N. Wear, J. E. Smith, H.-E. Andersen, B. J. Clough, W. B. Cohen, and D. M. Griffith. 2015. The US forest carbon accounting framework: stocks and stock change, 1990-2016.
79. Christman, M. C., D. H. Doctor, M. L. Niemiller, D. J. Weary, J. A. Young, K. S. Zigler, and D. C. Culver. 2016. Predicting the occurrence of cave-inhabiting fauna based on features of the earth surface environment. PLoS ONE 11:e0160408.
80. Weidner, E., and A. Todd. 2011. From the forest to the faucet: drinking water and forests in the US. Methods paper, Ecosystem Services and Markets Program Area, State and Private Forestry. USDA Forest Service.
81. U.S. Fish and Wildlife Service (2002) National wetlands inventory: a strategy for the 21st century. U.S. Fish and Wildlife Service, Washington
82. Crawford, D.L., R.W. Rohrbaugh, K.R. Aldinger, S. Barker, D.A. Buehler, J. Confer, C. Friis, J. Larkin, I. Lovette, J.D. Lowe, M. Piorkowski, K.V. Rosenberg, A. Roth, C. Smalling, P.B. Wood, R. Vallendaer. Spatio-temporal variation in the distribution of Golden-winged Warbler and Blue-winged Warbler as a function of climate; implications for management and conservation. In prep.
83. Iverson, L. R., A. M. Prasad, S. N. Matthews, and M. Peters. 2008. Estimating potential habitat for 134 eastern US tree species under six climate scenarios. Forest Ecology and Management 254:390–406.
84. Anderson, M.G., Barnett, A., Clark, M., Prince, J., Olivero Sheldon, A. and Vickery B. 2016. Resilient and Connected Landscapes for Terrestrial Conservation. The Nature Conservancy, Eastern Conservation Science, Eastern Regional Office. Boston, MA.
85. Sheldon, A. O., A. Barnett, and M. G. Anderson. 2015. A Stream Classification for the Appalachian Region. The Nature Conservancy, Eastern Conservation Science, Eastern Regional Office. Boston, MA.
86. Crawford, S., Whelan, G., Infante, D.M., Blackhart, K., Daniel, W.M., Fuller, P.L., Birdsong, T., Wieferich, D.J., McClees-Funinan, R., Stedman, S.M., Herreman, K., and Ruhl, P. 2016. Through a Fish's Eye: The Status of Fish Habitats in the United States 2015. National Fish Habitat Partnership.
87. DeWeber, J. T., & Wagner, T. (2014). Predicting Brook Trout Occurrence in Stream Reaches throughout their Native Range in the Eastern United States. Transactions of the American Fisheries Society, 144(1), 11-24.
88. Glick, P., B.A. Stein, & N.A. Edelson, editors. (2011). *Scanning the conservation horizon: A Guide to Climate Change Vulnerability Assessment*. National Wildlife Federation, Washington, D.C.
89. Klausmeyer, K. R., Shaw, M. R., MacKenzie, J. B., & Cameron, D. R. (2011). Landscape-scale indicators of biodiversity's vulnerability to climate change. *Ecosphere*, *2*(8).
90. Ackerly, D. D., Loarie, S. R., Cornwell, W. K., Weiss, S. B., Hamilton, H., Branciforte, R., & Kraft, N. J. (2010). The geography of climate change: Implications for conservation biogeography. *Diversity and Distributions*, *16*(3), 476-487.

91. Baettig, M. B., Wild, M., & Imboden, D. M. (2007). A climate change index: Where climate change may be most prominent in the 21st century. *Geophysical Research Letters*, *34*(1).
92. Williams, J. W., Jackson, S. T., & Kutzbach, J. E. (2007). Projected distributions of novel and disappearing climates by 2100 AD. *Proceedings of the National Academy of Sciences*, *104*(14), 5738-5742.
93. Diffenbaugh, N. S., & Giorgi, F. (2012). Climate change hotspots in the CMIP5 global climate model ensemble. *Climatic change*, *114*(3-4), 813-822.
94. Hamann, A., T. Wang, D.L. Spittlehouse, and T.Q. Murdock. 2013. A comprehensive, high-resolution database of historical and projected climate surfaces for western North America. Bulletin of the American Meteorological Society 94: 1307–1309.
95. Wang, T., Hamann, A., Spittlehouse, D. L., & Murdock, T. Q. (2012). ClimateWNA-High-resolution spatial climate data for western North America. *Journal of Applied Meteorology and Climatology*, *51*(1), 16–29.
96. IPCC 2013. Climate change 2013: the physical science basis. Contribution of working group I to the fifth assessment report of the intergovernmental panel on climate change.
